# Crystal structure of a constitutive active mutant of adenosine A_2A_ receptor

**DOI:** 10.1107/S2052252522001907

**Published:** 2022-03-17

**Authors:** Min Cui, Qingtong Zhou, Yueming Xu, Yuan Weng, Deqiang Yao, Suwen Zhao, Gaojie Song

**Affiliations:** a Shanghai Key Laboratory of Regulatory Biology, Institute of Biomedical Sciences and School of Life Sciences, East China Normal University, Shanghai 200241, People’s Republic of China; b Department of Pharmacology, School of Basic Medical Sciences, Fudan University, Shanghai 200032, People’s Republic of China; c State Key Laboratory of Oncogenes and Related Genes, Ren Ji Hospital, Shanghai Jiao Tong University School of Medicine, Shanghai 200127, People’s Republic of China; d iHuman Institute, ShanghaiTech University, Shanghai 201210, People’s Republic of China; e School of Life Science and Technology, ShanghaiTech University, Shanghai 201210, People’s Republic of China

**Keywords:** constitutive active mutants, adenosine A_2A_ receptors, crystal structures, hydrophilic interaction networks, molecular dynamics, G-protein-coupled receptors, agonists

## Abstract

The adenosine A_2A_ receptor (A_2A_AR) is a prototypical member of the class A subfamily of G-protein-coupled receptors (GPCRs) that is widely distributed in various tissues and organs of the human body, and participates in many important signal-regulation processes. We have previously summarized a common activation pathway of class A GPCRs in which a series of conserved residues/motifs undergo conformational change during extracellular agonist binding and finally induce the coupling of intracellular G protein. Through this mechanism we have successfully predicted several novel constitutive active or inactive mutations for A_2A_AR. To reveal the molecular mechanism of mutation-induced constitutive activity, we determined the structure of a typical mutant I92N complexed with the agonist UK-432097. The mutated I92N forms a hydrophilic interaction network with nearby residues including Trp^6.48^ of the CWxP motif, which is absent in wild-type A_2A_AR. Although the mutant structure is similar overall to the previously determined intermediate-state A_2A_AR structure (PDB ID 3qak) [Xu, Wu, Katritch, Han, Jacobson, Gao, Cherezov & Stevens (2011). *Science*, **332**, 322–327 ▸], molecular dynamics simulations suggest that the I92N mutant stabilizes the metastable intermediate state through the hydrophilic interaction network and favors the conformational transition of the receptor towards the active state. This research provides a structural template towards the special pharmacological outcome triggered by conformational mutation and sheds light on future structural or pharmaco­logical studies among class A GPCRs.

## Abstract 2

The structure of constitutive active mutant I92N reveals a hydrophilic network in the transmission-switch region of the adenosine A_2A_ receptor (A_2A_AR). The structure combined with molecular dynamics simulations suggests that the mutant I92N preserves the intermediate state in the presence or removal of an agonist, thus this sheds light on its higher basal activity.

## 1. Introduction

G-protein-coupled receptors (GPCRs) are a group of seven-transmembrane (7TM) proteins that can sense extracellular chemical/light/odor signals to transduce downstream cellular adaptors such as G proteins (Venkatakrishnan *et al.*, 2013 ▸; Yang *et al.*, 2021 ▸). The signal transduction is accomplished through binding of its agonist in the extracellular pocket that triggers conformational changes of the 7TM, which in turn creates enough space in the intracellular region to accommodate G protein binding (Weis & Kobilka, 2018 ▸; Rasmussen *et al.*, 2011 ▸). While the ligand–receptor binding modes are varied among different receptors, the transition pathways from a ligand-free inactive state to both agonist- and G-protein-bound active states are roughly similar on the intracellular side, and are characterized by a narrow inward movement of TM7 toward TM3 and a wide outward movement of TM6 in their cytoplasmic ends. In addition to the active and inactive snapshots determined by crystallography or cryo-EM, there are also a series of intermediate states during the conformational transition, which have been well illustrated by NMR studies (Ye *et al.*, 2016 ▸; Manglik *et al.*, 2015 ▸). The special role of the sodium-coordinating residues (such as Asp^2.50^ and Ser^3.39^) [Ballesteros–Weinstein numbering in superscript (Ballesteros & Weinstein, 1995 ▸)] on receptor stability and G-protein signaling has been comprehensively explored from the aspects of structural biology (Ballesteros & Weinstein, 1995 ▸; White *et al.*, 2018 ▸) and biophysics (Eddy *et al.*, 2018 ▸; Song *et al.*, 2019 ▸; Lee *et al.*, 2019 ▸), while the importance of other motif residues (such as CWxP, PIF and DRY) is yet to be determined. We have previously reported a common GPCR activation pathway that directly links the ligand-binding pocket with the G-protein-binding region (Zhou *et al.*, 2019 ▸). This common activation mechanism features the switching or repacking of dozens of paired residues within the intracellular half of the 7TM, including those that are conserved class A motifs (Thal *et al.*, 2018 ▸; Erlandson *et al.*, 2018 ▸). This mechanism is confirmed by designing constitutive active or inactive mutations within the pathway. Using A_2A_ adenosine receptor (A_2A_AR) as an example, we have functionally validated six mutations with increased basal activity (*i.e.* constitutive active mutations) and 15 mutations with decreased or abolished activity (*i.e.* constitutive inactive mutations) (Zhou *et al.*, 2019 ▸).

Abnormal function of A_2A_AR has been linked to neurodegenerative diseases such as Parkinson’s disease, Huntington’s disease, inflammation and coronary heart disease. Furthermore, A_2A_AR has been considered as a prototypical receptor in the GPCR structural biology field, and dozens of A_2A_AR structures with different types of ligands and/or adaptors have been determined. A_2A_AR in complex with an antagonist was crystallized in an inactive state (PDB ID 4eiy; Liu *et al.*, 2012 ▸), while these agonist-bound A_2A_ARs were mostly crystallized in the intermediate state (Xu *et al.*, 2011 ▸; Lebon *et al.*, 2015 ▸, 2011 ▸). Compared with the full active conformation that is acquired with the presence of G protein or mini-G protein (Carpenter *et al.*, 2016 ▸; Garcia-Nafria *et al.*, 2018 ▸), the intermediate state is within the receptor’s transition pathway from inactive state to active state. Given that these structures mainly focused on the ligands/effectors without touching function-related mutations, the understanding of mutation-induced receptor signaling transduction is not complete (Ballesteros & Weinstein, 1995 ▸). To investigate whether these constitutive active mutations are linked to unobserved conformational states of A_2A_AR, we tried to crystallize agonist-bound A_2A_AR in combination with different constitutive active mutations (Zhou *et al.*, 2019 ▸): I92^3.40^N, L95^3.43^A and I238^6.40^Y. Among them, I92^3.40^N was predicted to form amide-π interactions with Trp246^6.48^, while L95^3.43^A and I238^6.40^Y were thought to loosen the hydrophobic lock between Leu95^3.43^, Ile238^6.40^ and Val239^6.41^. All these mutations are hypothesized to favor the active conformation by rotating the intracellular half of TM6, which thus loosens the TM3–TM6 contacts to allow TM6 to move outward more easily to create enough space for recruiting downstream G protein.

## 2. Results

**Figure 1:**
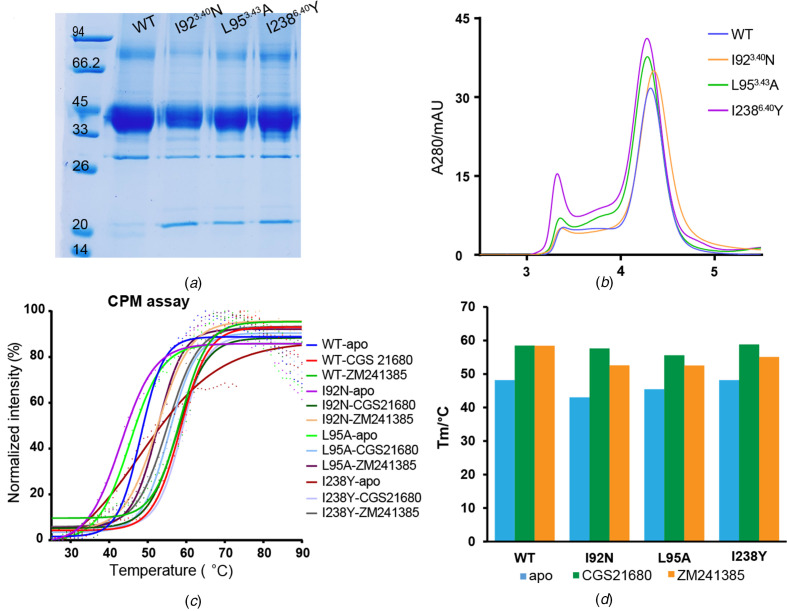
Purification and thermal-shift assay of A_2A_AR constitutive active mutants. (*a*) SDS–PAGE of purified WT and mutant A_2A_AR fusion proteins. (*b*) Size-exclusion chromatography plots (SECs) suggest that the A_2A_AR fusion proteins are mostly monomeric and of similar homogeneity. (*c*) Thermal-shift profiles and (*d*) melting temperature (*T*_m_) plots of A_2A_AR WT and mutants in the apo state or in complex with the agonist (CGS21680) or antagonist (ZM241385). In the thermal-shift assay, 500 m*M* NaCl was added in parallel to each experimental buffer for strict comparison since sodium is an allosteric effector for A_2A_AR.

All mutations were made based on a previous crystallized A_2A_AR construct with the third intracellular loop (ICL3) replaced with BRIL (Liu *et al.*, 2012 ▸) [referred to as wild type (WT) A_2A_AR hereafter, unless further mentioned]. These variants were expressed in insect cells and purified to similar homogeneity as WT A_2A_AR [Figs. 1 ▸(*a*) and 1 ▸(*b*)]. We firstly measured their thermal stabilities in the apo state or in complex with the agonist (CGS21680) or antagonist (ZM241385) by *N*-[4-(7-di­ethyl­amino-4-methyl-3-coumarinyl)­phenyl]­male­imide (CPM)-based thermal-shift assay [Fig. 1 ▸(*c*)]. Without the presence of ligands, all apo variants were relatively unstable with relatively lower melting temperatures. Notably, the WT A_2A_AR showed the best thermal stabilities in all conditions compared with these variants. Specifically, the I92^3.40^N and L95^3.43^A apo proteins each showed a significantly decreased stability (3–5°C) compared with the WT, whereas the decreases can be fully retrieved with the presence of CGS21680 but only partially retrieved by ZM241385 [Fig. 1 ▸(*d*)]. The results suggest that these mutations indeed alter the equilibrium of WT A_2A_AR and drive the receptor from inactive towards intermediate and finally to active state, and the metastable intermediate or active state can be stabilized by the agonists that favor the active state, whereas the antagonists that lean towards the inactive state are incom­patible with these mutations. Remarkably, mutant I238^6.40^Y showed a comparable melting temperature with WT in the apo state [Fig. 1 ▸(*d*)], suggesting that although Tyr238^6.40^ also destabilized the receptor via a conformational change towards the active state this may be compromised by its bulky aromatic side-chain which stabilized the local environment. We have previously summarized a similar mutagenesis strategy for thermal stability on a class B GPCR: glucagon-like peptide-1 receptor (Xu *et al.*, 2019 ▸).

Since the agonist performed better than the antagonist in thermal-shift assays (and also logically as described), we tried co-crystallization of all three variants with agonist CGS21680 but failed. We then tried co-crystallization with another agonist, UK-432097, which has similar potency to CGS21680 and was the first agonist that crystallized with WT A_2A_AR (Xu *et al.*, 2011 ▸). We successfully crystallized mutants I92^3.40^N and L95^3.43^A with UK-432097 but could only optimize the crystals of I92^3.40^N to a suitable size, and collected the data to 3.8 Å (Fig. 1 ▸, Table 1 ▸ and Fig. S1 of the supporting information). The structure was determined using a previous intermediate-state A_2A_AR structure (PDB ID 3qak; Xu *et al.*, 2011 ▸) as the search model. One asymmetric unit contains two molecules and these two are identical in the conformational state, herein we only refer to molecule A as the densities in molecule A are clearly better than in the other molecule.

**Figure 2:**
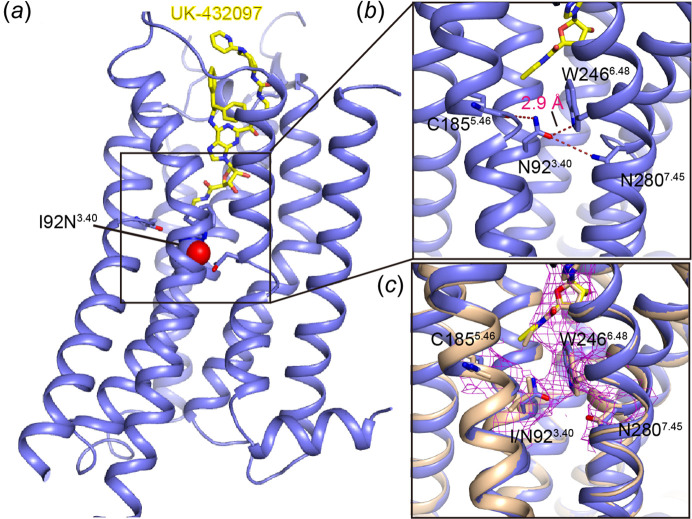
The I92^3.40^N mutant structure of A_2A_AR in complex with UK-432097. (*a*) The overall structure with agonist UK-432097 shown as yellow sticks and Asn92^3.40^ shown as spheres. (*b*) A zoomed-in view of the region around Asn92^3.40^ within the mutant structure. Hydrophilic interactions are marked with red dashed lines. (*c*) A superposition of the mutant structure with the intermediate-state WT structure (PDB ID 3qak). The electronic densities between Asn92 and Trp246 are shown at 2*F*_o_ − *F*_c_ of 1.0σ. In the WT structure the carbons are shown as light orange.

The I92^3.40^N–UK-432097 structure is similar overall to a previous intermediate A_2A_AR structure (Xu *et al.*, 2011 ▸) with a Cα r.m.s.d. of 0.46 Å, and is distinct from the inactive (Liu *et al.*, 2012 ▸) (Cα r.m.s.d. of 1.74 Å) or active (Carpenter *et al.*, 2016 ▸) (Cα r.m.s.d. of 1.61 Å) state structure. Similar to our previous prediction (Zhou *et al.*, 2019 ▸), in the variant structure, Asn92^3.40^ forms a hydrogen bond with Trp246^6.48^ [Figs. 2 ▸(*a*) and 2 ▸(*b*)]; such a hydrophilic interaction can also be suggested by the continued electronic densities between the two residues [Fig. 2 ▸(*c*)]. Meanwhile, the side chain of Asn92^3.40^ also forms a weak hydrogen bond with the carbonyl group of Cys185^5.46^, as well as an even weaker interaction with the side chain of Asn280^7.45^. All these residues are relatively far away from each other in the inactive state (Erlandson *et al.*, 2018 ▸), thus these residues and their local structures undergo conformational change and move together during receptor activation. Obviously, the above hydrophilic interactions are not possible in WT A_2A_AR with its endogenous Ile92^3.40^ residue [Fig. 2 ▸(*c*)]. Actually, Ile92^3.40^, well known as part of the P^5.50^I^3.40^F^6.44^ motif that triggers signaling initiation (Schönegge *et al.*, 2017 ▸; Wacker *et al.*, 2017 ▸; Zhou *et al.*, 2019 ▸), is located in an edge between the transmission switch and the hydrophobic lock in the structure, whereas in the inactive state it is involved more with the hydrophobic lock. Therefore, it is our estimation that the I92^3.40^N mutation disturbed the local environment in the apo (inactive) state, while this disharmony may be compromised through adding the agonist that induces departure of Asn92^3.40^ from the hydrophobic lock and formation of these hydrophilic interactions, these analyses are in line with the thermal-shift assay [Figs. 1 ▸(*c*) and 1 ▸(*d*)].

For A_2A_AR, the active state is roughly identical with the intermediate state in the transmission-switch region but distinct in the intracellular end of TM6, which moves further outward by >10 Å to accommodate G protein. The transition from intermediate to active state in the intracellular region requires the switch and new interactions formed by key residues Arg102^3.50^ and Tyr288^7.53^, as well as the residues in G protein. However, in the I92^3.40^N–UK-432097 structure we did not see further conformational change in the intracellular end of TM6 compared with the previous intermediate A_2A_AR structure. This is consistent with previous findings that full activation of a GPCR requires engagement of its downstream G protein, as validated in many receptors including A_2A_AR and β_2_AR (Nygaard *et al.*, 2013 ▸; Thal *et al.*, 2018 ▸; Eddy *et al.*, 2018 ▸; Ye *et al.*, 2016 ▸).

**Figure 3:**
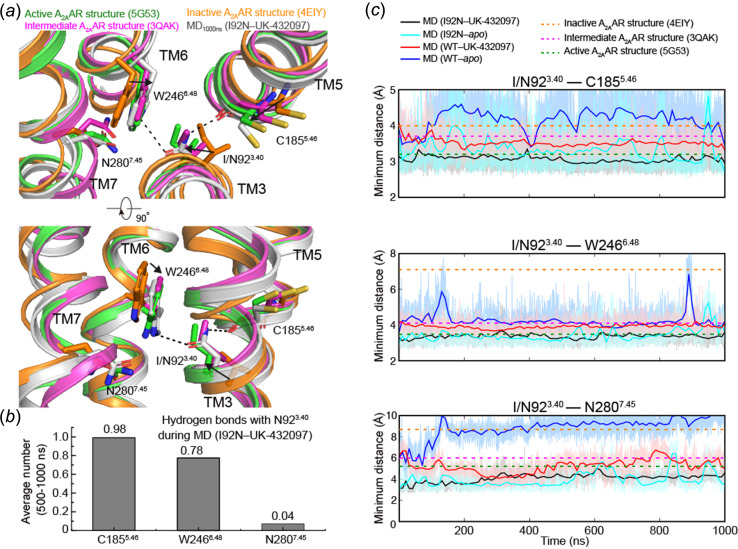
Conformational dynamics of the ligand-binding pocket in the MD simulations of WT A_2A_AR and its mutant I92N. (*a*), Structural comparison of the residues around Ile/Asn92^3.40^ between a representative MD snapshot and the released structures of A_2A_AR in different states. Inactive (antagonist bound), intermediate (agonist bound) and active (both agonist and G-protein bound) A_2A_AR structures are colored in orange, magenta and green, respectively (Liu *et al.*, 2012 ▸; Xu *et al.*, 2011 ▸; Carpenter *et al.*, 2016 ▸). (*b*) Statistics of hydrogen bonds between Asn92 and its surrounding residues during the last 500 ns MD simulation of UK-432097-bound A_2A_AR mutant I92N. (*c*) Representative distances between Ile/Asn92^3.40^ and its surrounding residues Cys185^5.46^, Trp246^6.48^ and Asn280^7.45^. Minimum distances were measured between non-hydrogen atoms for the selected two residues. Dashed horizontal lines indicate values for the released structure of A_2A_AR in different states (inactive state, orange; intermediate state, magenta; active state, green).

A crystal structure typically represents a single conformation of an individual protein, while it is known that GPCRs are very dynamic and multiple conformations are employed during their physiological events (Latorraca *et al.*, 2017 ▸). To further explore the dynamic events of the variant and the previous WT A_2A_AR intermediate structure, we performed all-atom molecular dynamics (MD) simulations to monitor how the I92^3.40^N mutation might affect the dynamics or conformation of A_2A_AR. All simulations including I92^3.40^N and WT together with UK-432097 or without UK-432097 (apo) were conducted on a 1 µs timescale (Figs. 3 ▸ and 4 ▸). The average r.m.s.d. of 2–4 Å (Cα) during these simulations indicates a reliable system for each trajectory (Fig. S2). Structural comparison among the inactive, intermediate and active structures of A_2A_AR reveals that the step-wise conformational change occurred in the residues centered at Ile92^3.40^ [Fig. 3 ▸(*a*)] up to final dense packing upon receptor activation, as seen from the decreasing inter-residue minimum distances [Fig. 3 ▸(*c*)]. When bound to UK-432097 (trajectory I92N–UK-432097), the mutated Asn92^3.40^ was mostly stabilized in its original position, which is identical to the active/intermediate states but distinct from the inactive state [Fig. 3 ▸(*a*)]. Quantitatively, Asn92^3.40^ preserves its hydrogen-bond interactions with Cys185^5.46^ and Trp246^6.48^ to percentages of 98% and 78%, respectively [Fig. 3 ▸(*b*)]. In the mutant simulation without UK-432097 (trajectory I92N–apo), the Asn92^3.40^–Cys185^5.46^ interaction is largely disrupted; in contrast, the Asn92^3.40^–Trp246^6.48^ interaction is well maintained at an early stage and fluctuation happens only during the second half of the timescale [Fig. 3 ▸(*c*)]. For the simulations of WT A_2A_AR, the minimum distances between Ile92^3.40^ and Trp246^6.48^/Cys185^5.46^/Asn280^7.45^ are also measured. The minimum distances of Ile92^3.40^–Trp246^6.48^ and Ile92^3.40^–Cys185^5.46^ are roughly stable during the simulation with UK-432097 (trajectory WT–UK-432097); in contrast, with removal of the agonist (trajectory WT–apo), both distances fluctuate and are apparently larger than those in the presence of UK-432097 [Fig. 3 ▸(*c*)]. The Ile/Asn92^3.40^–Asn280^7.45^ distance is not that sensitive overall compared with the other two pairs of distances. However, we can still see that the minimum Ile92^3.40^–Asn280^7.45^ distance is apparently larger on average in the simulation of WT–apo (without UK-432097) compared with WT–UK-432097, while for the Asn92^3.40^–Asn280^7.45^ distance we cannot differentiate between the simulations of I92N–UK-432097 and I92N–apo [Fig. 3 ▸(*c*)]. All these results indicate that, in addition to the agonist which drives the transition of the receptor from the inactive to the ntermediate/active state by forming multiple interactions with the pocket residues, I92^3.40^N also plays an essential role by disturbing the local environment and forming the hydrophilic linkages, which accompanies conformational change of the intracellular G-protein binding region.

**Figure 4:**
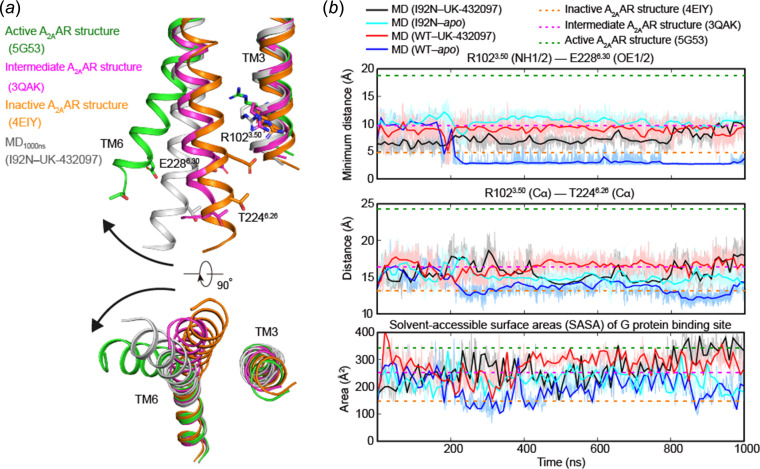
Conformational dynamics of the intracellular part of TM6 during MD simulations. (*a*) Structural comparison of the intracellular half of TM6 between a representative MD snapshot and the released structure of A_2A_AR in different states. Inactive, intermediate and active conformations are colored in orange, magenta and green, respectively (Liu *et al.*, 2012 ▸; Xu *et al.*, 2011 ▸; Carpenter *et al.*, 2016 ▸). All other TMs and ICLs are omitted for clarity. (*b*) Movements of TM6 during MD simulations: top, minimum distance between the charged non-hydrogen atoms of Arg102^3.50^ and Glu228^6.30^; middle, the Cα distance between Arg102^3.50^ and the intracellular tip of TM6 (Thr224^6.26^); bottom, the SASA of G-protein binding sites, which consists of Arg102^3.50^, Ala105^3.53^, Ile106^3.54^, Ile200^5.61^, Ala203^5.64^, Ser234^6.36^ and Leu235^6.37^. The interface areas were calculated by *FreeSASA* (Mitternacht, 2016 ▸). Dashed horizontal lines indicate values for the released structure of A_2A_AR in different states (inactive state, orange; intermediate state, magenta; active state, green).

At the intracellular region, the distinct performance of conformational dynamics between the WT and mutant receptors during MD simulations suggested a unique role played by Ile/Asn92^3.40^. Within all four types of trajectories, the minimum distances between the ionic lock residues [Arg102^3.50^ (NH1/2) and Glu228^6.30^ (OE1/2)] are far less than that of the active state (18.8 Å for PDB ID 5g53; Carpenter *et al.*, 2016 ▸). Nevertheless, while all other trajectories fluctuated between the inactive and intermediate states, WT–apo apparently returned back to the inactive state after 200 ns of simulation, judging from the steadily formed ionic lock as well as the Cα–Cα distance between Arg102^3.50^ and the first residue of TM6 (Thr224^6.26^). Consistently, the solvent-accessible surface area (SASA) of the G-protein binding site for the WT–apo snapshots is on average smaller than the other three. The trajectories of I92N–UK-432097 and I92N–apo are roughly similar, with far shorter distances of key residue pairs (Arg102^3.50^–Thr224^6.26^, Arg102^3.50^–Glu228^6.30^) [Fig. 4 ▸(*b*), top and middle] but much closer SASA of G-protein binding sites [Fig. 4 ▸(*b*), bottom] compared with those of the active structure. Such asynchronous events between the creation of intra­cellular cleft for G protein entering and further outward movement of the intracellular end of TM6 triggered by G-protein binding highlight the essential role of G-protein binding in receptor activation. Nevertheless, all these simulations suggest that although the I92^3.40^N mutant does not induce a full active state for A_2A_AR, it can preserve the intermediate state that is driven by the agonist through a hydrophilic interaction network; however, the WT receptor would return back to the inactive state within a short timescale once the agonist was removed.

A previous high-resolution structure of CGS21680-bound A_2A_AR has confirmed the presence of several waters within the ligand-binding pocket and sodium pocket (Lebon *et al.*, 2015 ▸). To determine the possibility that these waters may disturb the hydrophilic network revealed in our mutant structure, we performed further MD simulations with six water molecules modeled to the pockets of WT and I92N A_2A_AR (residues 2003–2008 of PDB entry 4ug2, chain B; Lebon *et al.*, 2015 ▸). In the additional mutant simulation, Asn92^3.40^ forms similar levels of hydrogen-bond interactions with Cys185^5.46^, Trp246^6.48^ and Asn280^7.45^ to the simulations without waters (Figs. 3 ▸ and S3), confirming that the hydrophilic network around Asn92^3.40^ can hardly be affected by these nearby solvents. Furthermore, both the WT structure (Lebon *et al.*, 2015 ▸) and its simulation revealed a weak hydrogen-bond interaction between Trp246^6.48^ and a nearby water, this interaction is mostly replaced by Asn92^3.40^ in the mutant simulation [Figs. S3(*b*)–S3(*d*)]. Interestingly, we noticed that during MD simulations water molecules can enter into the ligand-binding pocket and finally form a continuous water channel that is adjacent to the sodium pocket, even without the pre-existence of solvent within the ligand-binding pocket. Meanwhile, there are two key waters that consistently form hydrogen bonds with the pocket residues including Asp52^2.50^, Ser91^3.39^, Asn92^3.40^ and Asn280^7.45^ [Fig. S3(*a*)]. Although these waters are located close to the solvent modeled in the previous crystal structure (Lebon *et al.*, 2015 ▸), they are nevertheless quite dynamic and capable of water exchange during simulations (Fig. S4). Their dynamic nature may facilitate water rearrangement during conformational change and is consistent with a previous reference showing internal waters are critical for receptor activation (Yuan *et al.*, 2014 ▸).

## 3. Discussion

In this study we determined the A_2A_AR constitutive active mutant I92^3.40^N in complex with the agonist UK-432097 to a resolution of 3.8 Å. We identified that the mutation I92^3.40^N stabilizes a hydrophilic interaction network that preserves an intermediate state in the presence or removal of the agonist through MD simulations, whereas the WT receptor tends to move back to the inactive state without the presence of the agonist (Fig. S5). Our results indicated that both WT and mutant receptors can hardly equilibrate to the fully active conformation during the simulation in a 1 µs timescale, with or without agonist. This observation is consistent with the critical role of G-protein binding in receptor activation, as revealed in previous structural and dynamic studies (Carpenter *et al.*, 2016 ▸; Eddy *et al.*, 2018 ▸; Ye *et al.*, 2016 ▸). Alternatively, long-timescale accelerated/enhanced MD simulations have been developed to escape local energy minima and efficiently sample the full energy landscape (McRobb *et al.*, 2016 ▸). However, we are not able to conduct long-timescale simulations because of limited computational resources, thus how I92^3.40^N may affect the receptor conformation in long-timescale simulation still awaits further investigation.

The residues involved in the common activation pathway are partially conserved within class A GPCRs, *e.g.* Trp^6.48^ is located in a highly conserved CWxP motif, while the opposing position 3.40 is not very conserved but typically adopts a residue with a short side-chain to fit the highly condensed interaction network in the central region. Remarkably, several mutations on position 3.40 have been linked to dysfunctions or diseases, *i.e.* V509^3.40^A of thyrotropin receptor can cause non-autoimmune hyperthyroidism (Duprez *et al.*, 1994 ▸), I137^3.40^T of melanocortin receptor 4 can cause obesity (Gu *et al.*, 1999 ▸; Xiang *et al.*, 2006 ▸), S127^3.40^F of vasopressin V2 receptor can cause nephrogenic diabetes insipidus (Erdélyi *et al.*, 2015 ▸) and L125^3.40^R of rhodopsin can cause retinitis pigmentosa 4 (Dryja, 1992 ▸). Among these mutations, some may already change the local environment via their bulky side chains. These mutations may unbalance the activity of each receptor through either initiating the conformational transition (active) or disconnecting the transition linkage (inactive). Our study has laid the basis for understanding the mechanism for these disease-related mutations and can be effectively applied to future modeling studies for pharmacological or pathological purposes.

In summary, our research together with previous studies indicates the critical role of the transmission switch, and either agonist binding or specific mutations in the activation pathway may trigger receptor conformational change to achieve or maintain intermediate/active states. Our research provides a general template to understand the mutation-triggered conformational change and signal transduction though the combination of structural and computational biology, and highlights that mutation strategies may provide another routine to initiate signal transduction besides the classical agonist binding.

## 4. Materials and methods

### 4.1. A_2A_AR construct design, expression and purification

Human WT A_2A_AR gene has 412 residues. The crystallization construct replaced the ICL3 loop (residues Lys209–Gly218) with BRIL (thermostabilized apocytochrome b_562_ from *E. coli*) and cut off the C terminal after Ala316, which hindered the protein crystallization. The modified A_2A_AR gene was cloned in pFastBac-1 vector containing HA signal peptide, a FLAG epitope tag and a 3C protease cleavage site at the N terminus, and a 10× His-tag at the C terminus. Three mutations (I92^3×40^N, L95^3×43^A and I238^6×40^Y) were induced individually by overlap polymerase chain reaction (PCR) to form constitutively active mutations. Recombinant baculoviruses expressing A_2A_AR WT or mutants were prepared using the Bac-to-Bac system (Invitrogen). *Spodoptera frugiperda* 9 (Sf9) insect cells were grown in ESF921 medium; when the Sf9 cells density reached 2–3 × 10^6^ cells ml^−1^ they were infected by 1%(*v*/*v*) baculoviruses and harvested 48 h after infection. 1 l cells were collected by centrifugation, flash frozen in liquid nitro­gen and stored at −80°C until further use. After two washes of hypotonic buffer (10 m*M* HEPES pH 7.5, 10 m*M* MgCl_2_, 20 m*M* KCl with EDTA-free protease-inhibitor cocktail tablets) and three washes of high salt buffer (10 m*M* HEPES pH 7.5, 10 m*M* MgCl_2_, 20 m*M* KCl, 1 *M* NaCl with EDTA-free protease-inhibitor cocktail tablets), the cell pellets were collected and pre-treated with 4 m*M* theophylline (Sigma), 2.0 mg ml^−1^ iodo­acetamide (Sigma) and EDTA-free protease-inhibitor cocktail tablets. After incubation for 30 min, the cell membranes were solubilized by incubation in the presence of 50 m*M* HEPES, 500 m*M* NaCl, 1% *n*-do­decyl-β-d-maltoside (DDM, Anatrace) and 0.2% cholesterol hemisuccinate (CHS, Sigma) for 3 h at 4°C. The insoluble material was removed by centrifugation at 150 000*g* and the supernatant was added to 1 ml pure TALON resin (Clontech) and 20 m*M* imidazole, and left to rock slowly overnight at 4°C. The resin was washed with 4 × 10 column volumes of wash buffer (25 m*M* HEPES pH 7.5, 500 m*M* NaCl, 5% glycerol, 0.05% DDM, 0.01% CHS, 30 m*M* imidazole and 20 µ*M* UK-432097) and eluted with 3 ml elution buffer (25 m*M* HEPES pH 7.5, 500 m*M* NaCl, 5% glycerol, 0.025% DDM, 0.005% CHS, 300 m*M* imidazole and 100 µ*M* UK-432097). The elution was concentrated with a 100 kDa molecular-weight cut-off Amicon centrifugal ultrafiltration unit (Millipore).

### 4.2. Thermal-shift assay

CPM dye was dissolved in DMSO at 4 mg ml^−1^ as stock solution and diluted 20 times in CPM buffer [25 m*M* HEPES, pH 7.5, 500 m*M* NaCl, 5%(*v*/*v*) glycerol, 0.01%(*w*/*v*) DDM, 0.002%(*w*/*v*) CHS] before use. Then, 1 µl of diluted CPM was added to the same buffer with ∼0.5–2 µg receptor in a final volume of 50 µl. For receptors prepared for thermal-shift assay, no compound was added during purification and each compound was only added in each CPM buffer to a final concentration of 50 µ*M*. The thermal-shift assay was performed in a Rotor-Gene real-time PCR cycler (Qiagen). The excitation wavelength was 365 nm and the emission wavelength was 460 nm. All assays were performed over a temperature range from 25 to 85°C. The stability data were processed with *GraphPad Prism* (GraphPad Software, La Jolla, California, USA, https://www.graphpad.com/).

### 4.3. Crystallization

Purified A_2A_AR protein was cocrystallized with UK-432097 using lipid cubic phase (LCP) technology. The concentrated A_2A_AR was mixed with the lipid [10%(*w*/*w*) cholesterol, 90%(*w*/*w*) monoolein] using a 1:1.5(*v*:*v*) protein:lipid ratio to generate an LCP mixture, then each well on a 96-well plate was loaded with 50 nl of this mixture and overlaid with 800 nl of different precipitant solution. LCP plates were stored at room temperature (18–20°C). Diffracting quality crystals were grown in the condition 100 m*M* Tris pH 8.2, 30% PEG 400 and 0.4 *M* (NH_4_)_2_SO_4_. A_2A_AR–UK-432097 crystals were harvested using mesh grid loops (MiTeGen) and stored in liquid nitro­gen before use.

### 4.4. Data collection and model building

X-ray diffraction data were collected at the Japan synchrotron radiation SPring-8 facility on beamline 45XU (PILATUS 6M) with an automatic data-collection program. Diffraction data were collected with the 10 µm beam with 0.2 s exposures with an oscillation of 0.2° per frame. X-ray diffraction data were automatically processed with the program *KAMO* (Yamashita *et al.*, 2018 ▸), and indexed, integrated and scaled using *XDS* (Kabsch, 2010 ▸). The structure was solved by molecular replacement with *Phaser* (McCoy *et al.*, 2007 ▸) using the intermediate A_2A_AR structure (PDB ID 3qak) as the search model, then the fusion protein BRIL was manually modeled to the densities. Resulting model refinement and rebuilding were performed using *Phenix* (Adams *et al.*, 2010 ▸) and *Coot* (Emsley *et al.*, 2010 ▸). Statistics are provided in Table 1 ▸. The final 3D pictures were prepared with *PyMOL* (The PyMOL Molecular Graphics System, Version 2.0 Schrödinger, LLC).

### 4.5. Molecular dynamic simulations

Molecular dynamic simulations were performed by *Gromacs* 2020.1 (Abraham *et al.*, 2015 ▸). The WT A_2A_AR (UK-432097-bound A_2A_AR, PDB ID 3qak) and I92N mutant (crystal structure determined herein) were prepared and capped by the *Protein Preparation Wizard* (Schrödinger Suite 2019–2, Schrödinger, LLC, New York, USA, https://www.schrodinger.com) after the removal of fusion proteins. The missing loop in ICL3 was filled by *Prime* (Schrödinger Suite 2019–2). Two residues, Asp52^2.50^ and Asp101^3.49^, were deprotonated, while other titratable residues were left in their dominant state at pH 7.0. The apo receptor or its complex with UK-432097 was embedded in a bilayer composed of 201 POPC lipids and solvated with 0.15 *M* NaCl in explicitly TIP3P waters using *CHARMM-GUI Membrane Builder* (Wu *et al.*, 2014 ▸). The CHARMM36-CAMP force field (Guvench *et al.*, 2011 ▸) was adopted for protein, lipids and salt ions. The parameter of UK-432097 was generated using the *CHARMM General Force Field* (*CGenFF*) (Vanommeslaeghe *et al.*, 2010 ▸) program version 2.4.0. The particle mesh Ewald method (Darden *et al.*, 1993 ▸) was applied with a cut-off of 10 Å and the bonds involving hydrogen atoms were constrained using the *LINCS* algorithm (Hess, 2008 ▸). The MD simulation system was relaxed using the steepest descent energy minimization, followed by slow heating of the system to 310 K with restraints. The restraints were reduced gradually over 20 ns, with a simulation step of 1 fs. Finally, a 1000 ns production run without restraints was carried out, with a time step of 2 fs in the NPT ensemble at 310 K and 1 bar using a v-rescale thermo­stat (Bussi *et al.*, 2007 ▸) and a semi-isotropic Parrinello–Rahman barostat (Aoki & Yonezawa, 1992 ▸), respectively. The gmx hbond function within *Gromacs* was used to analyze hydrogen-bond occupancies (with applied criteria of donor–acceptor distance: 3.5 Å and 40° angle). The interface areas were calculated by *FreeSASA* (Mitternacht, 2016 ▸) using the Sharke–Rupley algorithm with a probe radius of 1.2 Å. Procedures and analysis for simulations with the presence of waters were identical, except that six waters were modeled to the initial models of mutant and WT according to their positions in PDB ID 4ug2 (residues 2003–2008 of chain B).

## Supplementary Material

Supporting information. DOI: 10.1107/S2052252522001907/mf5057sup1.pdf

PDB reference: adenosine A_2A_ receptor mutant I92N, 7ezc

## Figures and Tables

**Table 1:** Data-collection and refinement statistics Values for highest resolution shells are given in parentheses.

	I92^3.40^N–UK-432097
Data collection	
Space group	*C*2
Cell dimensions	
*a*, *b*, *c* (Å)	71.23, 175.70, 112.70
α, β, γ (°)	90, 91.21, 90
Resolution (Å)	47.43–3.80 (3.94–3.80)
Reflections (total/unique)	594392/13682
*R*_p.i.m._	0.14 (5.75)
CC_1/2_ †	0.99 (0.69)
〈*I*/σ(*I*)〉	10.46 (1.52)
Completeness (%)	99.9 (100)
Redundancy	43.4 (41.9)
	
Refinement	
*R*_work_/*R*_free_	0.284/0.316
R.m.s.d.	
Bond lengths (Å)	0.004
Bond angles (°)	0.641
Ramachandran plot (%)‡	93.5/6.1/0.4
PDB ID	7ezc

† CC_1/2_ = Pearson’s correlation coefficient between average intensities of random half data sets for each unique reflection.

‡ Residues in favored, accepted and outlier regions of the Ramachandran plot as reported by *MolProbity* (Williams *et al.*, 2018 ▸).
