# Supplementary material for: Crystal structure of a constitutive active mutant of adenosine A2A receptor
Source: IUCrJ. 2022 Mar 17;9(Pt 3):333–41. doi: 10.1107/S2052252522001907 (PMC9067115; doi:10.1107/S2052252522001907)
Supplement: Supplementary file 1 [file m-09-00333-sup1.pdf]

IUCrJ

Volume 9 (2022)

Supporting information for article:

Crystal structure of a constitutive active mutant of adenosine A2A receptor

Min Cui, Qingtong Zhou, Yueming Xu, Yuan Weng, Deqiang Yao, Suwen Zhao and Gaojie Song

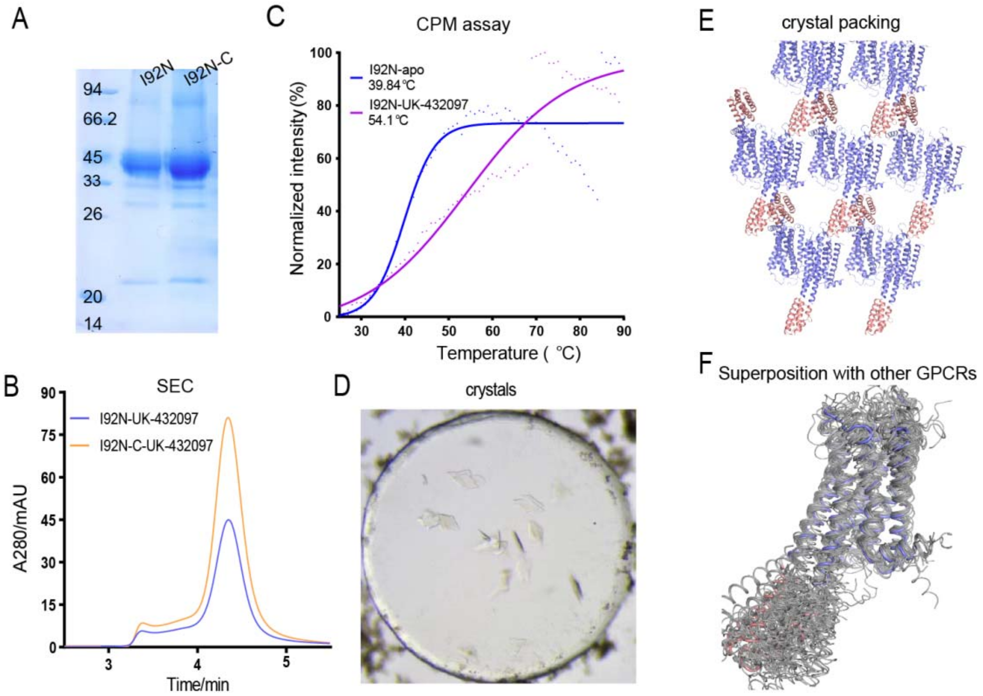

Figure S1 **Purification and crystallization of I92N mutant with UK-432097.** (**A-B**), SDS-PAGE and SEC profiles of purified mutant (I92N) and concentrated (I92N-C) I92N–UK-432097 complex. (**C**), Thermal-shift assay of mutant receptor in *apo* or in complex with UK-432097. Tm values are shown on the right. (**D**), Crystals of I92N–UK-432097 complex from LCP environment. (**E**), The lattice packing of I92N–UK-432097 structure. Receptors and BRILs are colored blue and red, respectively. (**F**), Comparison of I92N–UK-432097 with other GPCR structures crystallized with ICL3-BRIL fusion protein. Superposition was conducted on receptor by which showed variant orientations for ICL3-BRILs. The I92N mutant structure was colored blue (A_{2A}AR) and red (BRIL), whereas all other GPCRs were colored gray.

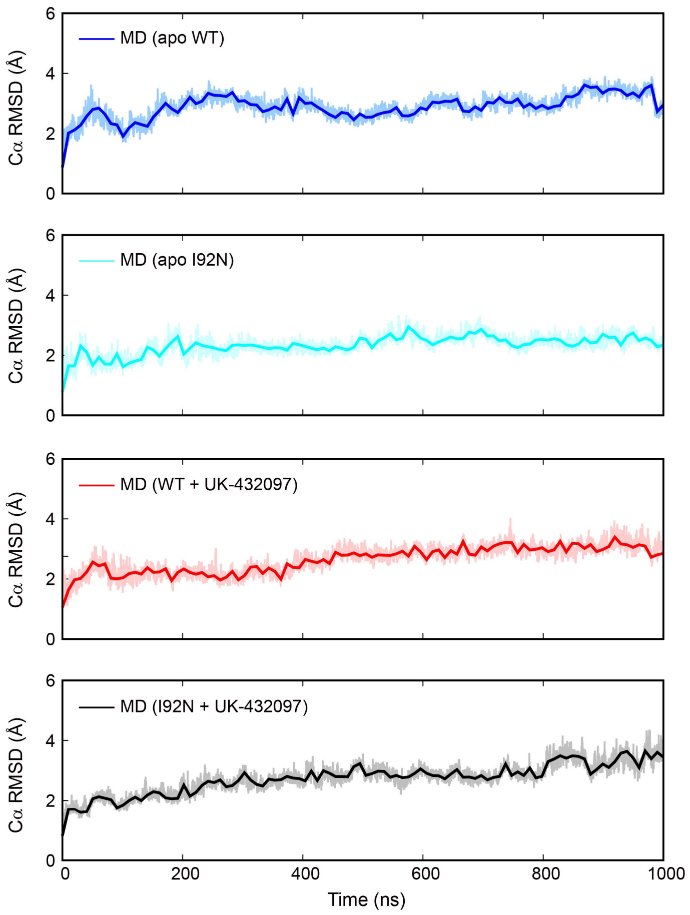

Figure S2 **Root mean square deviation (RMSD) of Cα positions of the WT A_{2A}AR and the mutant I92N in the MD simulations.** The MD snapshots based on WT model were superimposed on the crystal structures of WT A_{2A}AR (PDB code: 3QAK) using the Cα atoms, and the MD snapshots based on I92N were superimposed on current structure using the Cα atoms. The thick and thin traces represent moving averages and original, unsmoothed values, respectively.

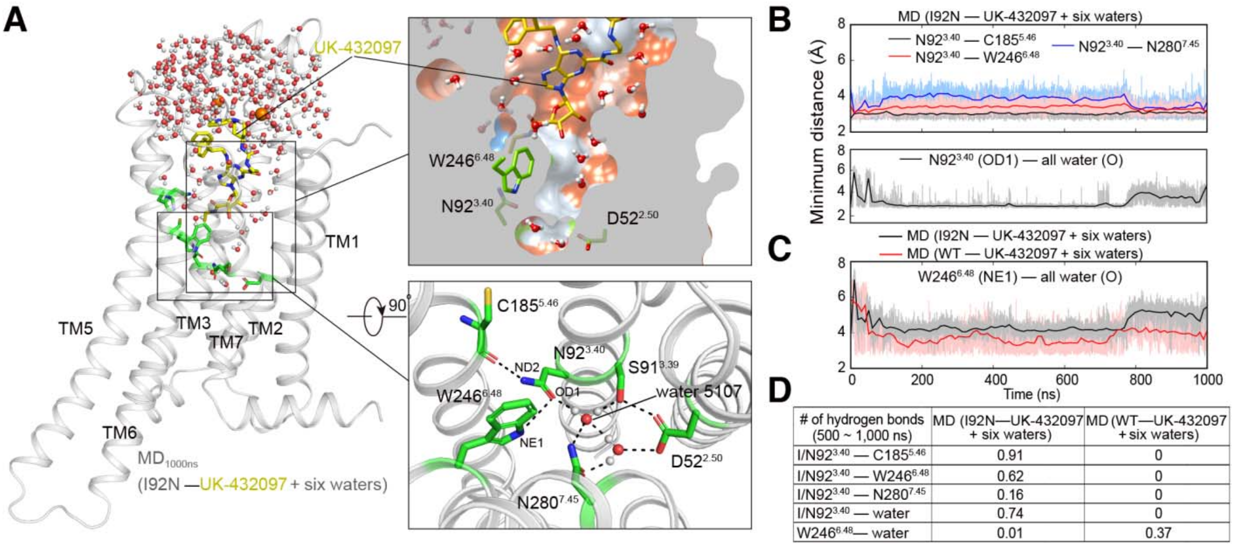

# of hydrogen bonds (500 ~ 1,000 ns)	MD (I92N—UK-432097 + six waters)	MD (WT—UK-432097 + six waters)
I/N92$^{3.40}$ — C185$^{5.46}$	0.91	0
I/N92$^{3.40}$ — W246$^{6.48}$	0.62	0
I/N92$^{3.40}$ — N280$^{7.45}$	0.16	0
I/N92$^{3.40}$ — water	0.74	0
W246$^{6.48}$— water	0.01	0.37

Figure S3 Water arrangement in the ligand-binding pocket during MD simulations with pre-existed six waters. (**A**), Final snapshot (1,000 ns) from the MD simulation of UK-432097-bound I92N mutant with six waters initially placed into the ligand-binding pocket according to the high-resolution CGS21680-bound crystal structure (residues 2003-2008 in chain B of 4UG2). The key residues around N92$^{3.40}$ and the water molecules in the ligand-binding pocket are shown in sticks. Close-up views of the ligand-binding pocket (right top) and water network around N92$^{3.40}$ (right bottom) show that a continuous water channel within the ligand-binding pocket as well as a stable water network connecting D52$^{2.50}$, S91$^{3.39}$, I92N$^{3.40}$ and N280$^{7.45}$. (**B**), Statistics of minimum distances between N92$^{3.40}$ and W246$^{6.48}$ or N280$^{7.45}$ or the closest water during simulation of UK-432097-bound I92N with pre-existed six waters. (**C**), Statistics of distances between W246$^{6.48}$ and the closest water during simulations of UK-432097-bound I92N or WT with pre-existed six waters. The relative far distances in I92N simulation compared to WT simulation indicated that W246$^{6.48}$ is less involved in hydrogen bond interaction with the closest water, as calculated in (D). (**D**), Statistics of hydrogen bonds between mentioned residues.

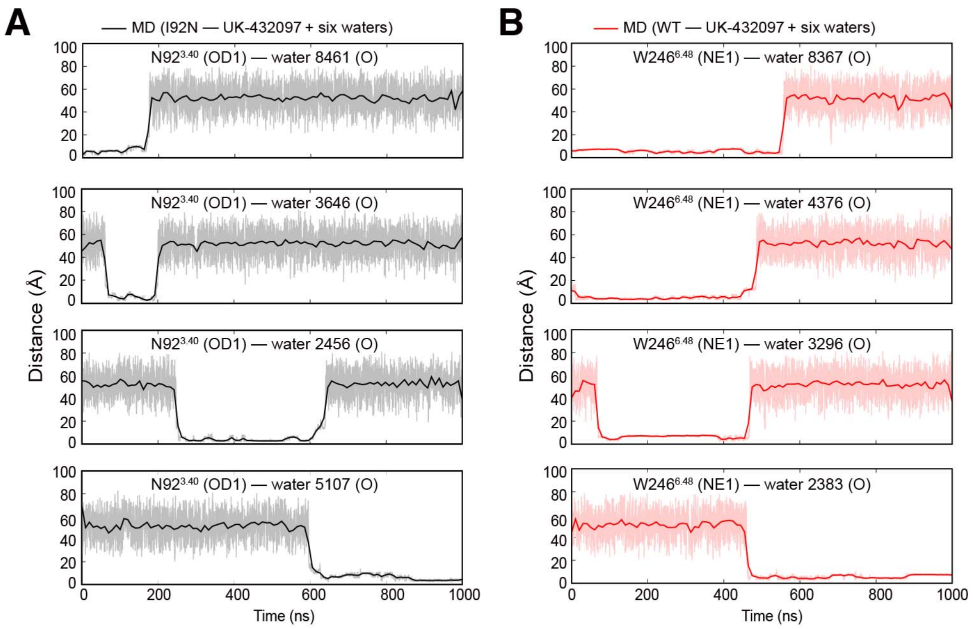

Figure S4 Water exchange in the ligand-binding pocket of UK-432097-bound I92N or WT $A_{2A}AR$ with pre-existed six waters in the MD simulations. (A), Minimum distances between the OD1 atom of $N92^{3.40}$ and the oxygen atoms of four representative water molecules (water 8461, 3646, 2456 and 5107) during MD simulation of UK-432097-bound I92N. (B), Minimum distances between the NE1 atom of $W246^{6.48}$ and the oxygen atoms of four representative water molecules (water 8367, 4376, 3296 and 2383) during MD simulation of UK-432097-bound WT $A_{2A}AR$. The six conserved waters were initially placed according to PDB 4UG2 (residues 2003-2008 of chain B).

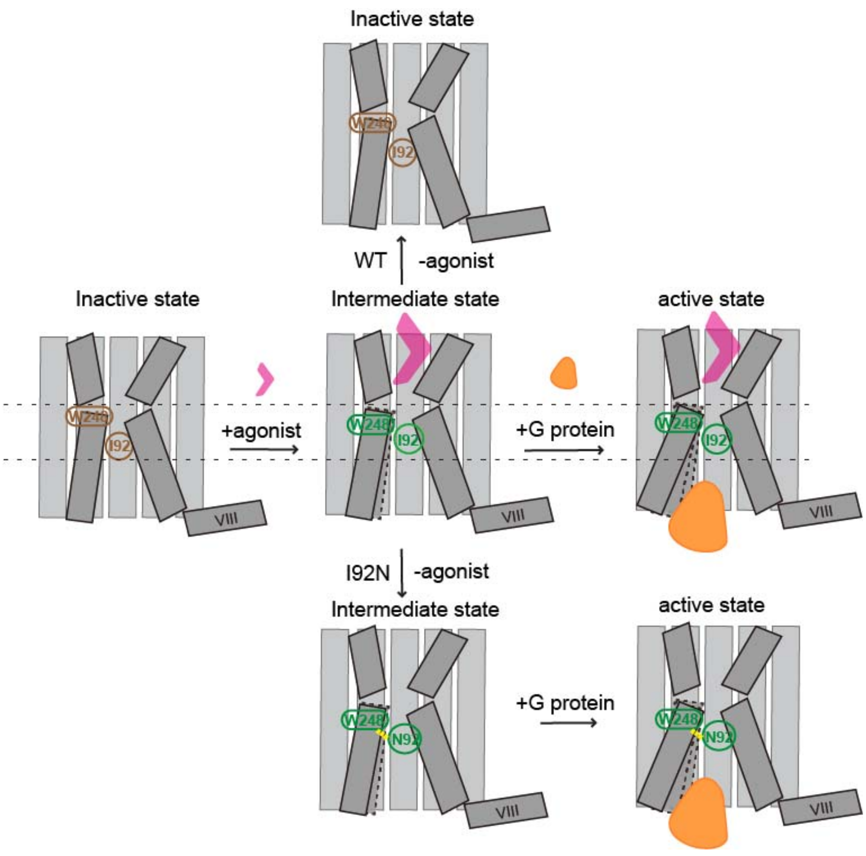

Figure S5 **The activation model for A_{2A}AR and I92N's effect.** Agonist like UK-432097 can trigger conformational change of A_{2A}AR from inactive state (left) toward intermediate state (middle) and the G protein can induce full activation of the receptor (right). When the agonist is removed the WT receptor can easily transit back to inactive state (top), whereas the mutant I92N can stabilize the receptor to the intermediate state to some extent (bottom), and readily accommodate the G protein for full activation (bottom right). This model explained the higher basal activity for I92N as well as other constitutively active mutations.

Table S1 Statistics of the calculated distances and surface area in MD simulations.

Measurements		MD (WT–apo)		MD (I92N–apo)		MD (WT–UK-432097)		MD (I92N–UK-432097)	
		0-1,000 ns	500-1,000 ns	0-1,000 ns	500-1,000 ns	0-1,000 ns	500-1,000 ns	0-1,000 ns	500-1,000 ns
Minimum distance (Å)	I/N92$^{3.40}$–C185$^{5.46}$	4.16 ± 0.49	4.21 ± 0.48	3.36 ± 0.51	3.30 ± 0.52	3.53 ± 0.24	3.50 ± 0.21	3.07 ± 0.26	3.02 ± 0.23
	I/N92$^{3.40}$–W246$^{6.48}$	4.25 ± 0.63	4.17 ± 0.67	3.46 ± 0.55	3.60 ± 0.65	3.92 ± 0.31	3.96 ± 0.32	3.37 ± 0.33	3.37 ± 0.31
	I/N92$^{3.40}$–N280$^{7.45}$	8.83 ± 1.22	9.43 ± 0.82	3.99 ± 0.91	4.35 ± 1.02	5.24 ± 0.85	5.61 ± 0.75	4.09 ± 0.54	4.27 ± 0.40
Distance (Å)	R102$^{3.50}$(NH1/2)–E228$^{6.30}$(OE1/2)	4.28 ± 2.77	2.96 ± 0.51	10.38 ± 1.15	10.23 ± 1.02	8.95 ± 1.27	8.96 ± 1.16	7.30 ± 1.33	7.79 ± 1.40
	R102$^{3.50}$(Cα)–T224$^{6.26}$(Cα)	13.91 ± 1.33	13.41 ± 1.02	15.15 ± 1.11	14.85 ± 0.94	16.41 ± 1.13	16.72 ± 0.87	15.64 ± 1.51	15.49 ± 1.49
Solvent-accessible surface area of G protein-binding sites (Å^2)*		199.44 ± 50.19	204.49 ± 37.77	227.16 ± 38.92	228.91 ± 44.50	278.48 ± 44.01	298.98 ± 29.81	265.18 ± 55.16	285.91 ± 54.92

*The solvent-accessible surface area of G protein-binding sites, which consists of R102$^{3.50}$, A105$^{3.53}$, I106$^{3.54}$, I200$^{5.61}$, A203$^{5.64}$, S234$^{6.36}$ and L235$^{6.37}$ were calculated by FreeSASA using the Sharke-Rupley algorithm with a probe radius of 1.2 Å.
